# Genetic and Biological Characterization of H3N2 Avian Influenza Viruses Isolated from Poultry Farms in China between 2019 and 2021

**DOI:** 10.1155/2023/8834913

**Published:** 2023-07-26

**Authors:** Jiqing Li, Guohua Deng, Jianzhong Shi, Yaping Zhang, Xianying Zeng, Guobin Tian, Yongping Jiang, Liling Liu, Huihui Kong, Hualan Chen

**Affiliations:** State Key Laboratory of Animal Disease Control and Prevention, Harbin Veterinary Research Institute, CAAS, Harbin, China

## Abstract

H3N2 influenza viruses not only cause seasonal epidemics in humans but also circulate widely in animals, posing a threat to both animal and human health. Our previous studies indicate that H3N2 avian influenza viruses (AIVs) are readily detected in live poultry markets (LPMs); however, the evolution and biological characteristics of the H3N2 viruses in poultry farms in China are unclear. In this study, we performed active surveillance and collected 49,135 samples from poultry farms. In total, 21 H3N2 AIVs were isolated and their genetics, receptor-binding properties, and replication in mice were evaluated. Phylogenetic analysis indicated that H3N2 AIVs harbor complicated gene constellations and have undergone extensive reassortment; the viruses derived their genes from 12 different hemagglutinin subtypes of duck viruses, including H1, H2, H4, H5, H6, H7, H8, H9, H10, H11, H12, and H14. The complicated gene constellations indicated that H3N2 viruses may have been introduced into poultry farms from different sources, but none have become dominant in poultry farms. Although the H3N2 AIVs possessed avian-type receptor-binding preference, most of the isolates could replicate without preadaptation and some of H3N2 viruses caused weight loss in mice. Notably, two H3N2 viruses acquired the PB2 627K mutation after a single round of replication in mice, suggesting similar mutations could occur if they replicated in humans. Overall, our study demonstrates that the H3N2 AIVs pose a potential threat to the public health and emphasizes the need for continued surveillance of H3N2 viruses in the both LPMs and poultry farms.

## 1. Introduction

Avian influenza viruses (AIVs) naturally circulate in waterfowl and sometimes cross the species barrier to infect humans and other mammals [1, 2]. AIVs are classified by the antigenic and genetic properties of their surface glycoproteins, hemagglutinin (HA) and neuraminidase (NA), into 18 HA subtypes and 11 NA subtypes [3, 4]. Sixteen HA subtypes and nine NA subtypes have been isolated from ducks, swans, and other waterfowl species [1]; H17N10 and H18N11 subtypes have only been identified from bats [5, 6]. The H1, H2, and H3 subtypes have successfully crossed the species barrier to infect humans and have caused at least four pandemics, with the H1N1 and H3N2 subtypes still actively circulating among humans. Wild waterfowl infected with AIVs are usually asymptomatic and exhibit weak immune responses [7]; therefore, different subtypes silently circulate and reassort in these natural reservoirs. As lessons from pandemics have shown, reassortment is the main mechanism by which novel viruses with pandemic potential may emerge [2].

Among all influenza A virus subtypes, the H3 subtype AIVs have been reported to have a diverse mammalian host range in addition to waterfowl [3, 4, 8]. In 1968, the H3 virus jumped from birds to humans and caused the H3N2 Hong Kong flu pandemic [9]; since then, it has been one of the causative pathogens of seasonal influenza in humans. The H3 subtype has also established stable circulation in other mammals, mainly, horses, pigs, dogs, and seals. In 1963, the first equine case of H3N8 infection was reported in the United States [10], and H3N8 has since become endemic in horses worldwide. In 1998, there were reports of severe respiratory diseases caused by H3N2 virus in breeding sows [11], and now the H3N2 viruses are wildly spreading among pig populations. In 2004, the first cases of canine influenza caused by H3N8 occurred in racing greyhounds [12], and in 2008, an avian-origin H3N2 canine virus was reported in South Korea [13]. The H3 subtype virus can even infect sea mammals; the first seal H3N3 virus was isolated in 1992, and an avian H3N8 virus infected seals in 2012, causing 162 deaths [14]. Additionally, H3 viruses have been isolated from mammals, such as cats, mink, and ferrets [4]. Moreover, human seasonal H3N2 and avian H3N2 spillovers into the swine population were observed [15, 16]. In 2010, H3N2 variant viruses (H3N2v) were detected with genes from avian, swine, and human viruses, and the 2009 H1N1 pandemic virus M gene [17]. Interspecies transmission of AIVs is an important factor in the evolution and ecology of influenza viruses [3, 4, 8]. Considering their wide host range and circulation in both avian and mammalian species, the H3 AIVs with various gene constellations provide abundant genome materials to generate a reassortant virus with pandemic potential.

The H3N2 subtype has been the main subtype circulating in avian species in China [18], and has undergone active reassortment with other influenza virus subtypes in live poultry markets (LPMs) [19–22], which could generate a virus with pandemic potential. We previously showed that H3N2 AIVs isolated from LPMs have complicated gene constellations, and some isolates can transmit among guinea pigs and ferrets [20]. Furthermore, we found that some H3N2 viruses recovered from ferrets obtained key mammalian adaptive mutations (i.e., HA Q226L or G228S), which changed the receptor-binding preference of the H3N2 virus from avian-type to human-type receptors, resulting in the efficient transmission of H3N2 AIVs between ferrets by respiratory droplets [23]. However, the diverse gene constellations and this biological characterization of H3N2 AIVs mainly represent the characteristics of viruses circulating in the ecosystem in LPMs, an artificial niche for easy reassortment between different AIV subtypes [24, 25], but not the natural ecosystem of H3 AIVs in animals. Hence, it is important to monitor the evolution of H3N2 AIVs in poultry farms. In this study, 21 H3N2 AIVs were identified from poultry farms through our routine surveillance between 2019 and 2021 in China, and their genetic characteristics, receptor-binding preference, and pathogenicity in mammals were investigated. Our findings will help us understand the characteristics of H3N2 AIVs in their natural hosts and the potential threat posed by recent H3N2 AIVs, which will expand our knowledge and ability to prevent and control, AIVs.

## 2. Materials and Methods

### 2.1. Ethics Statements and Facility

The study was performed following the recommendations in the Guide for the Care and Use of Laboratory Animals of the Ministry of Science and Technology of the People's Republic of China. The viral samples collected during active surveillance were processed in the enhanced biosafety Level 2 (BSL2+) facility at the Harbin Veterinary Research Institute of the Chinese Academy of Agricultural Sciences (HVRI, CAAS).

### 2.2. Virus Isolation and Identification

Through our routine active surveillance of poultry farms in China between February 2019 and April 2021, a total of 21 H3N2 subtype AIVs were isolated. Cloacal and tracheal swabs from the same animal were combined as one sample and preserved in phosphate-buffered saline (PBS) containing 2,000 U/mL penicillin and 2,000 *µ*g/mL streptomycin. To isolate viruses, the samples were centrifuged at 6,000 r/min for 3 min and then the supernatant was inoculated into 9- to 11-day-old embryonated chicken eggs and cultured at 37°C for 48 hr. Allantoic fluid collected from positive samples was subjected to the hemagglutinin inhibition (HI) assay with homemade H3 subtype-specific antisera to identify the H3 subtype, and the NA subtype was confirmed by Sanger sequencing. All viruses were biologically cloned three times by limiting dilution in embryonated specific-pathogen-free (SPF) eggs. Virus stocks were amplified in SPF chicken eggs and harvested allantoic fluids were preserved at −80°C.

### 2.3. Sequence and Phylogenetic Analyses

The genomes of the H3N2 viruses (GISAID accession numbers: EPI2257143-EPI2257310) were extracted for genetic and phylogenetic analyses in this study. Viral RNA (vRNA) extraction was performed by using a TIANamp Virus RNA Kit (Tiangen, Beijing, China) according to the manufacturer's protocol, and then cDNA was synthesized by reverse transcription with the Uni12 primer (AGCAAAAGCAGG) and amplified by PCR with primers complementary to the conserved promoter and noncoding region of each gene segment (primers available on request). Sequencing was performed by using the BigDye Terminator Cycle Sequencing Kit and analyzed on an ABI 3500xL genetic analyzer (3500xL Genetic Analyzer, USA).

The nucleotide sequences were edited by using the SeqMan module of the DNAStar and aligned by using the MAFFT in the PhyloSuite (v1.2.2) software package [26]. The MegAlign module of the DNAStar software package was used to calculate the sequence identity. The phylogenetic trees of the genes were analyzed by using the neighbor-joining method with a bootstrap value of 1,000. A ≥ 95% cutoff was used to categorize gene segments into different groups in the phylogenetic trees. The sequences used in the trees were downloaded from GenBank (https://www.ncbi.nlm.nih.gov/genbank/) or GISAID (https://platform.epicov.org/epi3/start). In brief, a tree built with all available sequences (full-length only and removing the identical sequences) was generated, and sequences in the respective branches were selected to build the final tree based on virus epidemiological information (*Supplementary 1*).

### 2.4. Receptor-Binding Specificity Analysis

Receptor-binding properties were analyzed by using a solid-phase binding assay with two artificially synthesized glycopolymers: *α*-2,3-siaylglycopolymer (Neu5Aca2- 3Galb1-4GlcNAcb1-pAP- (para-aminophenyl-) alpha-polyglutamic acid (a-PGA)) (avian-type receptor) and *α*-2,6-sialylglycopolymer (Neu5Aca2-6Galb1-4GlcNAcb1-pAP- (para-aminophenyl-) alpha-polyglutamic acid (a-PGA)) (human-type receptor) as described previously [27]. First, the viruses were purified through 30% sucrose in PBS. Then, the purified viruses with a series of twofold dilutions were incubated with a plate coated with the two different glycopolymers at 4°C overnight. The next day, the plate was washed with cold PBS and then immobilized with 4% formalin. After the plate was again washed five times with PBST (PBS containing 0.1% Tween 20), chicken antiserum against the indicated viruses was added to the wells and incubated at 37°C for 1 hr. Then, the plate was washed and subsequently incubated with a horseradish peroxidase (HRP)-conjugated goat-anti-chicken antibody (Sigma–Aldrich, St. Louis, MO, USA) for 1 hr at 37°C. The plate was subjected to color development with O-phenylenediamine (Sigma–Aldrich, St. Louis, MO, USA). The absorbance was measured at 490 nm.

### 2.5. Study in Mice

The replication and virulence of H3N2 AIVs were evaluated in groups of eight 6-week-old female BALB/c mice (Vital River Laboratories, Beijing, China). The mice were lightly anesthetized with CO_2_, then intranasally inoculated with 10^6^ 50% egg infective dose (EID_50_) of the indicated H3N2 viruses in a volume of 50 *μ*L as previously described [28]. The mock group was inoculated with 50 *μ*L of PBS. On Day 3 postinoculation (p.i.), three of the eight infected mice in each group were euthanized, and their organs, including nasal turbinates, lungs, kidneys, spleens, and brains were collected for virus titration in eggs. The virus titers were calculated by using the Reed and Muench [29] method. The remaining five mice in each group were monitored daily for weight loss and mortality for 14 days.

### 2.6. Statistics Analysis

Raw data from the samples were converted to the logarithmical scale before further analysis. Statistical significance between different groups was assessed by using the Unpaired *t*-test in GraphPad Prism (v8) software. Assume both populations have the same standard deviation (SD). *P* < 0.05 was considered statistically significant.

## 3. Results

### 3.1. Virus Isolation

To characterize H3N2 AIVs in their natural hosts rather than the artificial ecosystem created by humans in LPMs, we collected 49,135 samples from poultry farms in 27 provinces, autonomous regions, and municipalities of China, during our routine surveillance from February 2019 to April 2021. With an isolation rate of 0.33%, we isolated 163 virus strains, belonging to the H1, H3, H4, H5, H6, H9, and H11 subtypes. Among the AIVs, 21 H3N2 viruses were isolated, including 4 chicken viruses and 17 duck viruses (Table 1), indicating that the H3N2 AIVs were mainly circulating in ducks. The H3N2 viruses were isolated from 10 of 27 sampling regions (i.e., Guangdong, Guangxi, Fujian, Jiangxi, Hunan, Sichuan, Guizhou, Anhui, Jiangsu, and Chongqing), indicating that H3N2 AIVs are mainly circulating in the southern regions of China.

### 3.2. Phylogenetic Analyses of the HA and NA Genes

**Figure 1:**
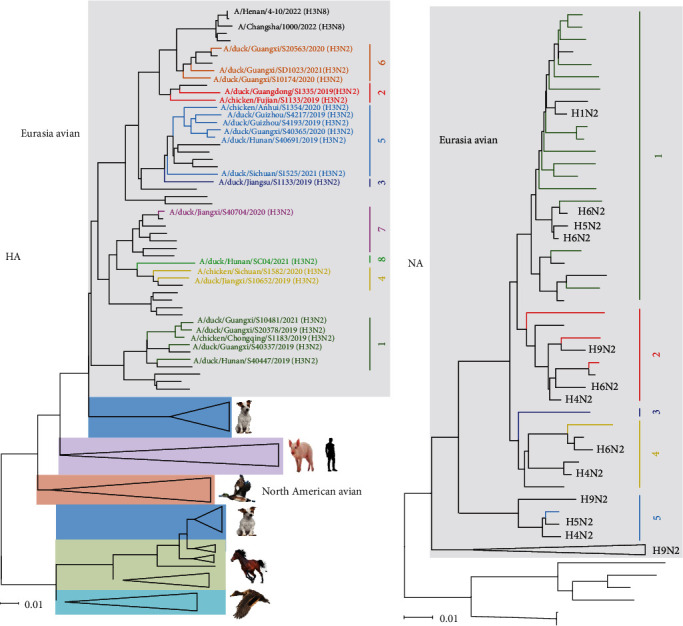
Phylogenetic analysis of the surface genes of H3N2 avian influenza viruses. The phylogenetic tree was generated by using the neighbor-joining method and the MEGA 7.0 software package, with 1,000 bootstrap replicates. Sequences with a nucleotide identity of more than 95% were categorized into the same group: (a) phylogenetic tree of the HA gene; (b) Phylogenetic tree of NA gene. The viruses isolated in this study are colored in the phylogenetic trees: viruses in black were downloaded from available databases; viruses not belonging to Eurasian avian lineage were compressed. The scale bar indicates the number of nucleotide substitutions per site.

To elucidate the genetic relationship of the H3N2 AIVs in poultry farms of mainland China, the genomes of the 21 isolates were compared with available H3 viruses downloaded from databases. The evolutionary relationship of the 21 HA genes is presented in a tree constructed by using the neighbor-joining method (Figure 1(a)). The HAs of the 21 H3N2 AIVs belong to the Eurasian avian lineage, which exhibits a clear difference from the canine, equine, swine, and human H3 viruses. A high degree of genetic diversity was observed among the 21 HAs with nucleotide similarity between 85.7% and 98.9%, leading to the HAs being divided into eight groups. Surprisingly, the HA gene of three isolates in Group 6 (Figure 1(a)), A/duck/Guangxi/S10174/2020, A/duck/Guangxi/S20563/2020, and A/duck/Guangxi/SD1023/2021, was closely related to that of two human H3N8 isolates, A/Henan/4-10/2022 and A/Changsha/1000/2022.

The NA genes of the 21 H3N2 viruses in this study belong to the Eurasian lineage. With a nucleotide similarity between 88.1% and 99.1%, the N2 genes were classified into five groups in the tree. Group 1 contains most of the viruses (*n* = 15), Group 2 contains three viruses, whereas Groups 3, 4, and 5 each contain only one virus (Figure 1(b)). Of note, Group 3, with only one virus (A/duck/Guangdong/S1335/2019), formed a new branch with no known origin, since BLAST analysis indicated that there was no NA gene with a nucleotide similarity above 95% in the databases. From the phylogenetic tree (Figure 1(b)), frequent exchanging of the N2 gene between H3 and other subtype viruses was observed. A duck H9N2 virus, A/duck/Fujian/11.26_FZHX0195-O/2018, shared an extremely high nucleotide similarity (99.08%) with A/duck/Jiangsu/S1133/2019 NA in Group 2 (Figure 1(b) and *Supplementary 2*), indicating that H9N2 can directly acquire the NA gene from H3N2 AIVs or *vice versa*, although the H3N2 NA was distinct from the main NA branch of H9N2 viruses (Figure 1(b)). Some N2 genes were categorized into the same groups containing H1 and H4, H5, and H6 subtype viruses (Figure 1(b)).

### 3.3. Phylogenetic Analysis of the Internal Genes

**Figure 2:**
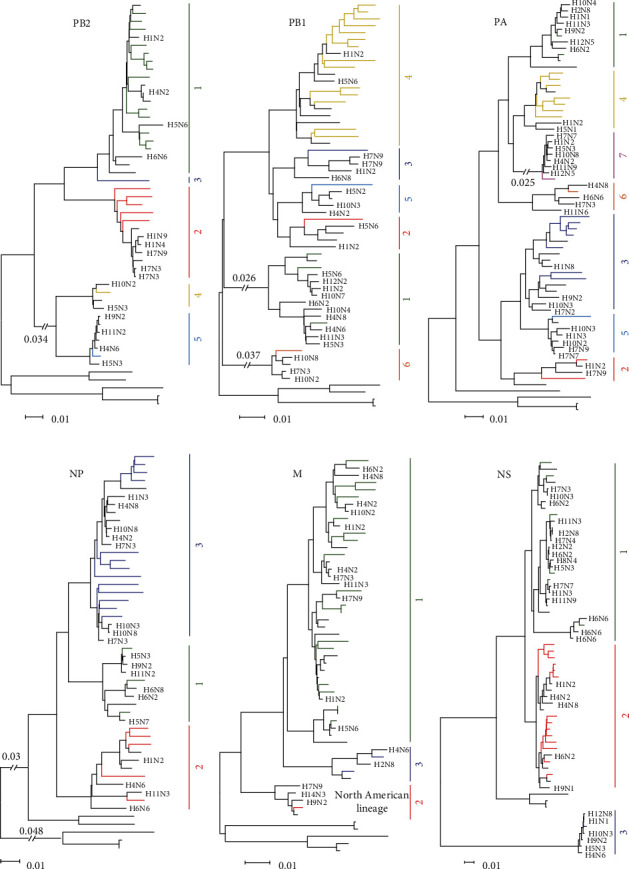
Phylogenetic analysis of the PB2 (a), PB1 (b), PA (c), NP (d), M (e), and NS (f) genes of H3N2 avian influenza viruses. The phylogenetic tree was generated by using the neighbor-joining method and the MEGA 7.0 software package, with 1,000 bootstrap replicates. Sequences with a nucleotide identity of more than 95% were categorized into the same group. The viruses isolated in this study are colored in the phylogenetic trees; viruses in black were downloaded from available databases. The scale bar indicates the number of nucleotide substitutions per site.

The internal genes of the 21 H3N2 viruses exhibited considerable diversity, with the basic polymerase 2 (PB2), basic polymerase 1 (PB1), acidic polymerase (PA), nucleoprotein (NP), matrix (M), and nonstructural protein (NS) genes of the viruses sharing nucleotide similarities of 86.6%–99.3%, 89.3%–98.9%, 88.8%–99%, 90.2%–98.7%, 92.2%–99.8%, and 70.6%–99.6%, respectively. In general, the PB1 and PA genes of H3N2 AIVs were more diverse than the other four genes. The PB2, PB1, and PA genes were classified into five, six, and seven groups, respectively, whereas the NP, M, and NS genes each contained three groups (Figure 2). All the genes of the 21 H3N2 viruses belong to the Eurasian lineages; however, the one exception was that the M gene of one virus in Group 2 (A/duck/Guangxi/S40337/2019), which originated from viruses in the North American lineage (Figure 2(e) and *Supplementary 2*). Like the surface genes, all the internal genes of the viruses originated from different bird species, especially ducks (*Supplementary 2*). In addition, the internal genes underwent frequent reassortment with multiple subtypes. In general, the internal genes of H3N2 AIVs are similar to those of H1, H4, H5, H6, H7, H10, and H11 subtypes viruses (Figure 2), which are commonly detected in poultry farms and in LPMs [19–21]. Genes of H9 subtype viruses were found to be clustered into the same groups formed by the PB2, PA, NP, M, and NS genes of H3N2 AIVs (Figures 2(a), 2(c), 2(d), 2(e), and 2(f) and *Supplementary 2*). Genes of H12 subtype viruses were detected within the same groups in the PB1, PA, and NS trees (Figures 2(b), 2(c), and 2(f) and *Supplementary 2*). Genes of H2 subtype viruses were found within the classified groups of PA, M, and NS genes (Figures 2(c), 2(e), and 2(f) and *Supplementary 2*). Moreover, the NS gene of an H8N4 virus and the M gene of an H14N3 virus were found within the NS and M gene groups formed by AIVs (Figures 2(e) and 2(f) and *Supplementary 2*). In summary, the internal genes of the H3N2 AIVs were clustered into groups containing genes from as many as 12 HA subtype viruses, namely, H1, H2, H4, H5, H6, H7, H8, H9, H10, H11, H12, and H14 subtype viruses. Based on this genomic diversity, the 21 H3N2 AIVs were categorized into 19 genotypes, each genotype containing one virus, except for Genotypes 6 and 14, which contained two viruses each (Table 1).

### 3.4. Molecular Characterization

The cleavage sites of all 21 HAs contain only one basic amino acid (R), which implies that all the H3N2 isolates are low-pathogenic AIVs [24, 30]. Amino acid substitutions at many positions of HA are critical for pathogenicity, receptor binding, and the host range of AIVs. Therefore, we analyzed the amino acid sites in HA that influence the receptor-binding preference, including 138 (H3 numbering, used hereafter), 155, 158, 159, 160, 186, 189, 190, 192, 193, 218, 225, 224, 226, and 228 [23, 31–44]. Avian virus signatures were observed at 13 of these 15 positions, with the exceptions being positions 159 and 193 (Table 2). At position HA 159—a site involved in the adaptation of viruses in swine [42]—one virus contained G, two viruses contained S, and the other 18 viruses contained N. At position HA 193––a site that affects the receptor-binding specificity of AIVs [41]––one virus harbored G, nine viruses harbored S, and the remaining 11 viruses harbored N, suggesting a potential change in receptor-binding specificity. For the NA protein, we did not observe the deletion at positions 63–65, which can confer mammalian adaptation to H9N2 viruses [45].

Many sites in the internal genes of AIVs are associated with pathogenicity and transmission in mammals. Sequence analysis indicated that all 21 viruses contain avian virus signatures in many of these sites, including 271T, 590G, 591Q, 627E, 648L, and 701D in PB2; 269S, 207K, 436Y, 622G, and 677T in PB1; 224S, 356K, and 515T in PA; 286A, 357Q and 437T in NP; and 30D, 156D, and 215A in M1 [38, 46–56]. However, a few mammalian adaptive amino acid substitutions were detected in some genes (Table 2). In the PB2 gene, 292V and 588V, which play important roles in the transmission and adaptation of H7N9 and H10N8 viruses [48, 57], were detected in two and one virus isolates, respectively (Table 2). Surprisingly, all 21 isolates contained PA 383D, which is associated with increased polymerase activity in human cells [46]; 20 of the 21 isolates harbor NS1 42S, which is associated with the pathogenicity of H5 and H1 viruses in mammals [58, 59]. In addition, one isolate contained M2 31N, which is indicative of resistance to adamantine and rimantadine [60]. In summary, at most of the sites associated with mammalian adaptation, the H3N2 AIVs maintained avian signatures, but some viruses did acquire a few mammalian adaptative mutations [61]. These data indicate that the H3N2 AIVs may exhibit a mammalian-adaptative phenotype.

### 3.5. Receptor-Binding Specificity

**Figure 3:**
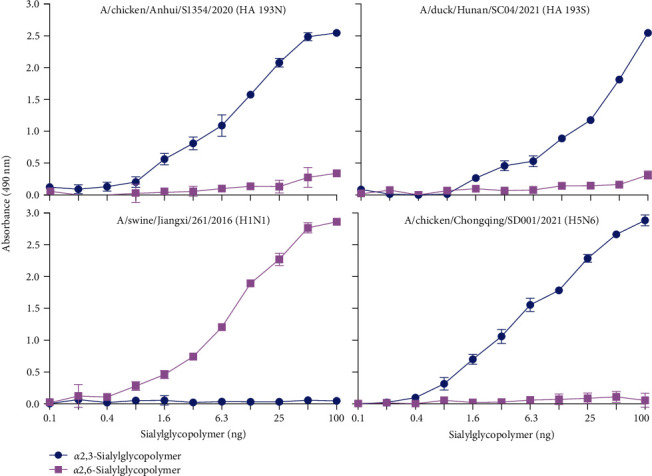
Receptor-binding specificity of H3N2 representative viruses. Binding affinity for avian-type and human-type receptors was analyzed by using two different glycans (*α*-2,6-siaylglycopolymer, pink; *α*-2,3-siaylglycopolymer, blue) the assay was performed in triplicate; error bars indicate standard deviations.

The essential prerequisite for AIVs to cross the species barrier from avian to human is the recognition of *α*-2,6-linked sialic acids (SAs) (human-type receptors). Molecular analysis indicated that most of the sites that affect receptor-binding preference were conserved, except Positions 159 and 193 in HA, which did not significantly change the antigenicity of the H3N2 viruses (*Supplementary 3*). Given that most recent H3N2 AIVs possess N at Position 159, but exhibit amino acid polymorphism at Position 193, the role of HA N193S in receptor-binding preference was investigated. Two H3N2 isolates, with N or S at Position 193 in HA, were selected for evaluation of receptor-binding preference with two synthesized glycopolymers. As shown in Figure 3, swine A/swine/Jiangxi/261/2016 (H1N1) and avian A/chicken/Chongqing/SD001/2021 (H5N6) preferentially bound *α*-2,6-linked sialic acids and *α*-2,3-linked sialic acids (avian-type receptors), respectively. Although the mutation HA N193S slightly reduced that binding affinity for human-type receptors, both A/chicken/Anhui/S1354/2020 (HA 193N) and A/duck/Hunan/SC04/2021 (HA 193S) exhibited avian-type receptor-binding specificity. These data suggest that all the H3N2 AIVs maintained their avian-type receptor-binding specificity.

### 3.6. Pathogenicity of the H3N2 Viruses in Mice

**Figure 4:**
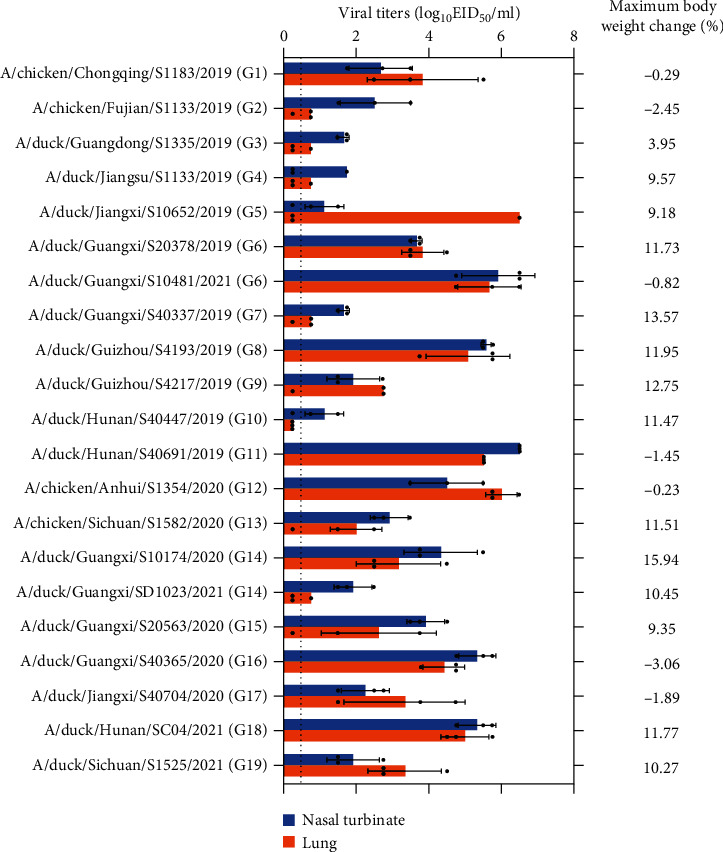
Replication and pathogenicity of H3N2 viruses in mice. Groups of eight mice were intranasally inoculated with 10^6^ EID_50_ of the indicated viruses. Three mice from each group were euthanized on Day 3 p.i. and the remaining five were observed for body weight changes for 2 weeks. Virus titers in organs were determined in eggs. The error bars represent standard deviations, and the dashed line indicates the lower limit of virus detection.

Low-pathogenic AIVs usually cause no signs of disease or mild disease in avian species [3, 4]. However, low-pathogenic AIVs may cause severe disease or even death in humans [62]. To evaluate the pathogenicity of the H3N2 AIVs in mammals, we examined the replication and virulence of all 21 H3N2 AIVs in BALB/c mice. All the viruses replicated in the nasal turbinates and lungs of the mice, with titers ranging from 10^1.1^ EID_50_/mL to 10^6.5^ EID_50_/mL and 10^0.75^ EID_50_/mL to 10^6.0^ EID_50_/mL, respectively, with the exception of one virus (A/duck/Hunan/S40447/2019) in Genotype 10, which did not replicate in the lungs of mice (Figure 4). No virus was recovered from the other organs tested (data are not shown). Seven viruses caused body weight loss, ranging from −0.23% to −3.06% (Figure 4), whereas 12 viruses caused body weight increases, ranging from 3.95% to 15.94%, over the 14-day observation period. These data indicate that H3N2 AIVs can replicate in the respiratory system of mammals without preadaptation and some of them could cause mild disease in mammals.

### 3.7. The Role of PB2 E627K in the Mammalian Adaptation of H3N2 Viruses

Since some of the 21 H3N2 viruses replicated without preadaptation and caused body weight loss in mice, we next asked whether these viruses could acquire mutations that facilitate mammalian adaptation. Sanger sequencing analysis of viral genomes extracted from infected mice revealed that two viruses, A/chicken/Anhui/S1354/2020 (S1354) and A/duck/Guangxi/S40365/2020 (S40365), acquired a dominant PB2 E627K mutation after replication in mice, suggesting a critical role for PB2 627K in their mammalian adaptation.

**Figure 5:**
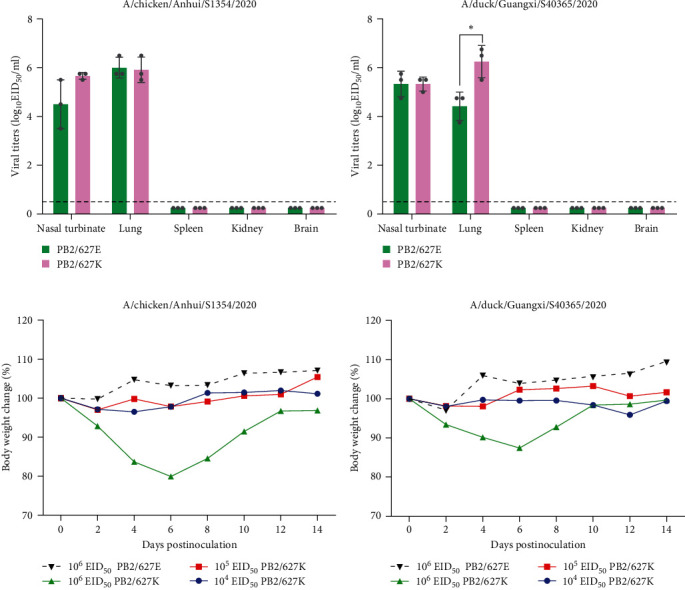
Replication and pathogenicity of H3N2 viruses with PB2 627E or 627K in mice: (a and b) groups of three mice were intranasally infected with 10^6^ EID_50_ of the indicated viruses, the mice were euthanized on Day 3 p.i. and the brains, nasal turbinates, spleens, kidneys, and lungs were collected for titration in eggs; (c and d) groups of five mice were intranasally inoculated with the indicated doses of the test viruses and body weight changes were observed for 2 weeks. The error bars represent standard deviations, and the dashed line indicates the lower limit of virus detection  ^*∗*^*P* < 0.05.

To further confirm the role of PB2 E627K mutation, we purified two viruses recovered from infected mice, acquiring mouse-adapted S1354 (maS1354) and mouse-adapted S40365 (maS40365), which contain 627K in PB2. Then we performed a comparative study of viruses with PB2 627E or PB2 627K in mice. Groups of eight mice were intranasally inoculated with various doses of indicated viruses, and their replication and virulence were monitored for 14 days. The replicative ability of maS1354 was comparable to that of S1354 in mice, as there was no significant difference between the titers in mice infected with the paired viruses (Figure 5(a)). The replicative ability of maS40365 was slightly higher than that of S40365 in mice; the titer in the lungs of mice infected with maS40365 627K was significantly higher than that of mice infected with S40365 (Figure 5(b)). The maximum body weight loss of mice inoculated with 10^6^ EID_50_ of maS1354 or maS40365 was −20.06% and −12.59%, respectively (Figures 5(c) and 5(d)). For mice inoculated with 10^6^ EID_50_ of S1354 or S40365, the maximum body weight loss was −0.23% and −3.06%, respectively (Figures 5(c) and 5(d)). The results indicate that the virus harboring PB2 627K was more virulent than the virus with PB2 627E in mice at the dose of 10^6^ EID_50_.

## 4. Discussion

In this study, we investigated the genetic and biological characteristics of 21 H3N2 AVIs isolated from poultry farms in China between 2019 and 2021. Consistent with the epidemiology data of H3N2 viruses in LPMs [19–21, 63], the H3N2 AIVs in poultry farms have undergone frequent reassortment and formed complicated genotypes. Phylogenetic analysis revealed that the H3N2 AIVs in poultry farms harbor similar genes to as many as 12 of the other 15 avian-origin HA subtype viruses, indicating extensive reassortment between H3N2 and other subtype viruses. The 21 H3N2 viruses formed 19 genotypes, revealing the considerable capacity of H3N2 viruses as an important gene pool of AIVs in poultry species. All the genes of the 21 H3N2 AIVs originated from the Eurasian lineage, except for one virus, which contained the M gene from North American wild ducks. Additionally, we identified a new N2 branch without a known origin. Although these viruses maintained avian-type receptor-binding preference and did not acquire a key mammalian adaptive mutation in HA, most of the isolates replicated well in mice without preadaptation, and two of them acquired PB2 627K after a single round of replication in mice. Our data thus reveal that the H3N2 AIVs pose a potential threat to humans and continued active surveillance in poultry farms is as important as that in LPMs.

We observed that H3N2 AIVs share similar genes with 12 HA subtype viruses, including the H1, H2, H4, H5, H6, H7, H8, H9, H10, H11, H12, and H14 subtypes. However, similarities with the genes of H13, H15, and H16 subtype AIVs, which are rarely isolated and mainly maintained in gulls [64–66], were not observed. The considerable capacity of H3N2 AIVs to tolerate genes from other subtype AIVs highlights their potential to generate a reassortant that could cause a pandemic. AIVs could jump from birds to humans after acquiring internal genes from H9N2 viruses, as demonstrated by human cases of H7N9, H5N6, H10N3, and H10N8 virus infection [25, 62, 67–69]. In 2022, two young boys were infected with a triple reassortant H3N8, which harbored the surface genes from H3 subtype AIVs and internal genes from avian H9N2 AIVs [70]. In the present study, we found that H3N2 AIVs tolerate PB2, PA, NP, M, NS, and N2 genes from H9N2 viruses. Although most H9N2 AIVs predominantly circulate in the chickens and most H3N2 viruses circulate in the ducks [18], the frequent isolation of H3N2 AIVs from the chickens strongly implies that the H3N2 AIVs could reassort with H9N2 viruses and generate a virus that poses a threat to humans.

A receptor-binding preference for human-type receptors is one of the key factors that facilitates the jump of AIVs from avian species to humans. Molecular analysis indicated that all the positions around the receptor-binding pocket were conserved, except position 193. Stevens et al. [71] demonstrated that the mutation HA K193R significantly increased the binding of an H5N1 virus to *α*-2,6-linked SAs. Medeiros et al. [43] reported that HA K193S significantly decreased the binding of HA from equine H3N8 viruses to chicken and sheep erythrocytes. Moreover, sequences analysis indicated that 47.3% of avian H3N2 viruses bear 193N, whereas only 1.4% of human H3N2 viruses bear 193N. These facts imply that HA N193S could alter the receptor-binding preference; however, our solid binding analysis revealed that HA N193S only slightly decreased the binding affinity for *α*-2,3-linked SAs. According to the findings of Medeiros et al. [43] the double mutations HA N193S and I194L induce the binding of human H3 to human-type receptors. Therefore, another mutation would be necessary to alter the receptor-binding preference of H3N2 AIVs. Our previous study found that the H3N2 AIVs easily acquire the mammalian adaptive mutation Q226L or G228S, which switches H3N2 AIVs to human-type receptor preference, resulting in efficient transmission in ferrets [23]. In our present study, we did not detect any HA mutation in viruses recovered from H3N2-infected mice. Xu et al. [72] investigated the distribution of the SAs in ferrets and found that abundant *α*-2,6-linked SAs were distributed in the respiratory tract of these animals [72, 73], whereas, both *α*-2,3-linked SAs and *α*-2,6-linked SAs were distributed in the respiratory tract of mice [74]. The relatively low abundance of *α*-2,6-linked SAs in mice may explain why the H3N2 AIVs did not acquire HA mutations that confer increased human-type receptor-binding affinity.

In our study, many H3N2 isolates replicated well in the nasal turbinates and lungs of infected mice, however, they caused only mild or no body weight loss, which is consistent with our previous study [20]. The pathogenicity of the influenza virus is dependent on both viral factors and the host immune system. The low pathogenicity may be attributed to the lacking of critical mammalian adaptive amino acid substitutions in viral genes (Table 2). PB2 is another key factor for the pathogenicity and mammalian adaptation of AIVs. Two amino acid mutations in PB2, E627K and D701N, are crucial for the replication and transmission of AIVs in mammals. PB2, E627K or D701N have emerged in many mammalian-adapted AIVs, such as H5, H7, and H9 AIVs [38, 45, 51, 57, 75]. For H3N2 viruses, the human and swine viruses typically harbor PB2 627K, whereas avian viruses contain the avian signature PB2 627E. Anthony et al. [14] found that seal H3N8 viruses naturally harbor PB2 701N. Yu et al. [76] reported that a mouse-adapted H3N2 avian virus contained PB2 701N. In contrast, in our study, PB2 627K was observed in viruses recovered from H3N2 AIV-infected mice. Although both PB2 627K and 701N are found in mammal-adapted AIVs, PB2 627K are more common than PB2 701D in human isolates. Liang et al. [77] demonstrated that the acquisition of PB2 627K is driven by the low polymerase activity attributed to PA, and PB2 701N will solely emerge in ANP32A knock-out mice. Of note, PB2 627K has emerged in human-infecting H3N8 AIVs [70]. This ready acquisition of PB2 627K, together with our previous finding that two mutations HA (226L or 228S) can switch H3N2 AIVs to human-type receptor specificity, strongly indicate that H3N2 AIVs pose a potential threat to humans.

In summary, here we genetically and biologically characterized H3N2 AIVs isolated from poultry farms. Our data indicate that the gene diversity of H3N2 AIVs in poultry farms is as complicated as that of H3N2 AIVs in LPMs. Notably, some H3N2 AIVs readily obtain the mammal-adapted mutation PB2 E627K in infected mice. Taken together, our findings emphasize the need for continued surveillance of H3N2 AIVs.

## Figures and Tables

**Table 1:** The H3N2 avian influenza viruses isolated from poultry farms in China.

No.	Strain	Location	Host	Date	Groups								Genotype
				(Year/month)	HA	NA	PB2	PB1	PA	NP	M	NS	
1	A/chicken/Chongqing/S1183/2019	Chongqing	Chicken	2019/3	1	1	1	1	1	1	1	1	G1
2	A/chicken/Fujian/S1133/2019	Fujian	Chicken	2019/3	2	2	2	2	2	2	1	1	G2
3	A/duck/Guangdong/S1335/2019	Guangdong	Duck	2019/3	2	3	3	3	2	2	1	1	G3
4	A/duck/Jiangsu/S1133/2019	Jiangsu	Duck	2019/4	3	1	2	6	5	3	1	1	G4
5	A/duck/Jiangxi/S10652/2019	Jiangxi	Duck	2019/2	4	2	1	4	1	3	1	2	G5
6	A/duck/Guangxi/S20378/2019	Guangxi	Duck	2019/4	1	1	1	4	3	3	1	2	G6
7	A/duck/Guangxi/S10481/2021	Guangxi	Duck	2021/2	1	1	1	4	3	3	1	2	G6
8	A/duck/Guangxi/S40337/2019	Guangxi	Duck	2019/12	1	1	1	4	4	3	2	2	G7
9	A/duck/Guizhou/S4193/2019	Guizhou	Duck	2019/10	5	4	1	4	3	3	1	2	G8
10	A/duck/Guizhou/S4217/2019	Guizhou	Duck	2019/10	5	1	2	4	4	3	1	2	G9
11	A/duck/Hunan/S40447/2019	Hunan	Duck	2020/12	1	1	4	1	3	1	1	1	G10
12	A/duck/Hunan/S40691/2019	Hunan	Duck	2019/11	5	2	2	5	3	3	3	2	G11
13	A/chicken/Anhui/S1354/2020	Anhui	Chicken	2020/4	5	1	2	4	3	3	1	2	G12
14	A/chicken/Sichuan/S1582/2020	Sichuan	Chicken	2020/5	4	1	1	4	6	3	3	2	G13
15	A/duck/Guangxi/S10174/2020	Guangxi	Duck	2020/5	6	1	1	4	4	2	1	2	G14
16	A/duck/Guangxi/SD1023/2021	Guangxi	Duck	2021/2	6	1	1	4	4	2	1	2	G14
17	A/duck/Guangxi/S20563/2020	Guangxi	Duck	2020/6	6	1	1	4	4	3	1	2	G15
18	A/duck/Guangxi/S40365/2020	Guangxi	Duck	2020/11	5	1	1	4	3	3	1	2	G16
19	A/duck/Jiangxi/S40704/2020	Jiangxi	Duck	2020/12	7	5	5	1	7	1	1	3	G17
20	A/duck/Hunan/SC04/2021	Hunan	Duck	2021/1	8	1	1	4	4	2	1	2	G18
21	A/duck/Sichuan/S1525/2021	Sichuan	Duck	2021/4	5	1	1	4	4	3	1	1	G19

**Table 2:** Key molecular markers of the H3 viruses in this study.

No.	Virus name	Amino acid^a^						
		HA		PB2		PA	M1	NS1
		159	193	292	588	383	31	42
1	A/chicken/Chongqing/S1183/2019	N	S	I	A	D	V	S
2	A/chicken/Fujian/S1133/2019	N	N	I	A	D	V	S
3	A/duck/Guangdong/S1335/2019	N	N	I	A	D	V	S
4	A/duck/Jiangsu/S1133/2019	N	N	I	A	D	V	S
5	A/duck/Jiangxi/S10652/2019	N	S	I	A	D	V	S
6	A/duck/Guangxi/S20378/2019	N	S	I	A	D	V	S
7	A/duck/Guangxi/S10481/2021	N	S	I	A	D	V	S
8	A/duck/Guangxi/S40337/2019	N	S	I	A	D	V	S
9	A/duck/Guizhou/S4193/2019	N	N	I	V	D	V	S
10	A/duck/Guizhou/S4217/2019	N	N	V	A	D	V	S
11	A/duck/Hunan/S40447/2019	N	S	V	A	D	V	S
12	A/duck/Hunan/S40691/2019	N	N	I	A	D	V	S
13	A/chicken/Anhui/S1354/2020	N	N	I	A	D	V	S
14	A/chicken/Sichuan/S1582/2020	N	S	I	A	D	V	S
15	A/duck/Guangxi/S10174/2020	G	N	I	A	D	V	S
16	A/duck/Guangxi/SD1023/2021	N	N	I	A	D	V	S
17	A/duck/Guangxi/S20563/2020	S	N	I	A	D	V	S
18	A/duck/Guangxi/S40365/2020	N	N	I	A	D	V	S
19	A/duck/Jiangxi/S40704/2020	N	N	I	A	D	V	A
20	A/duck/Hunan/SC04/2021	S	S	I	A	D	V	S
21	A/duck/Sichuan/S1525/2021	N	N	M	A	D	V	S

^a^Only amino acids that conferred mammalian adaptation or drug resistance are shown.

## Data Availability

Data are available in the article's supplementary material.

## Supplementary Materials

Supplementary 1 Accession number of the reference sequences in the phylogenetic tree.

Supplementary 2 Phylogenetic trees of the genes of H3N2 AIVs. The phylogenetic tree, HA (a), NA (b), PB2 (c), PB1 (d), PA (e), NP (f), M (g), and NS (h), was generated by using the neighbor-joining method and the MEGA 7.0 software package, with 1,000 bootstrap replicates. Sequences with a nucleotide identity of more than 95% were categorized into the same group. The viruses isolated in this study are colored in the phylogenetic trees; viruses in black were downloaded from available databases. The scale bar indicates the number of nucleotide substitutions per site.

Supplementary 3 Antigenic analysis of H3N2 AIVs isolated from 2019 to 2021 in China.

## References

[1] Dowdle W. R., Davenport F. M., Fukumi H. (1975). Orthomyxoviridae. *Intervirology*.

[2] Subbarao K., Katz J. (2000). Avian influenza viruses infecting humans. *Cellular and Molecular Life Sciences CMLS*.

[3] Webster R. G., Bean W. J., Gorman O. T., Chambers T. M., Kawaoka Y. (1992). Evolution and ecology of influenza A viruses. *Microbiological Reviews*.

[4] Wille M., Holmes E. C. (2020). The ecology and evolution of influenza viruses. *Cold Spring Harbor Perspectives in Medicine*.

[5] Tong S., Zhu X., Li Y. (2013). New world bats harbor diverse influenza A viruses. *PLoS Pathogens*.

[6] Tong S., Li Y., Rivailler P. (2012). A distinct lineage of influenza A virus from bats. *Proceedings of the National Academy of Sciences*.

[7] Kida H., Yanagawa R., Matsuoka Y. (1980). Duck influenza lacking evidence of disease signs and immune response. *Infection and Immunity*.

[8] Olsen B., Munster V. J., Wallensten A., Waldenström J., Osterhaus A. D. M. E., Fouchier R. A. M. (2006). Global patterns of influenza A virus in wild birds. *Science*.

[9] Kawaoka Y., Krauss S., Webster R. G. (1989). Avian-to-human transmission of the PB1 gene of influenza A viruses in the 1957 and 1968 pandemics. *Journal of Virology*.

[10] Waddell G. H., Teigland M. B., Sigel M. M. (1963). A new influenza virus associated with equine respiratory disease. *Journal of the American Veterinary Medical Association*.

[11] Zhou N. N., Senne D. A., Landgraf J. S. (1999). Genetic reassortment of avian, swine, and human influenza A viruses in American pigs. *Journal of Virology*.

[12] Crawford P. C., Dubovi E. J., Castleman W. L. (2005). Transmission of equine influenza virus to dogs. *Science*.

[13] Song D., Kang B., Lee C. (2008). Transmission of avian influenza virus (H3N2) to dogs. *Emerging Infectious Diseases*.

[14] Anthony S. J., St. Leger J. A., Pugliares K. (2012). Emergence of fatal avian influenza in New England harbor seals. *mBio*.

[15] Nelson M. I., Vincent A. L. (2015). Reverse zoonosis of influenza to swine: new perspectives on the human–animal interface. *Trends in Microbiology*.

[16] Kida H., Shortridge K. F., Webster R. G. (1988). Origin of the hemagglutinin gene of H3N2 influenza viruses from pigs in China. *Virology*.

[17] Centers for Disease Control and Prevention (CDC) (2012). Update: Influenza A (H3N2)v transmission and guidelines—five states, 2011. *MMWR: Morbidity and Mortality Weekly Report*.

[18] Yang J., Yang L., Zhu W., Wang D., Shu Y. (2021). Epidemiological and genetic characteristics of the H3 subtype avian influenza viruses in China. *China CDC Weekly*.

[19] Zou S., Tang J., Zhang Y. (2020). Molecular characterization of H3 subtype avian influenza viruses based on poultry-related environmental surveillance in China between 2014 and 2017. *Virology*.

[20] Guan L., Shi J., Kong X. (2019). H3N2 avian influenza viruses detected in live poultry markets in China bind to human-type receptors and transmit in guinea pigs and ferrets. *Emerging Microbes & Infections*.

[21] Liu T., Huang Y., Xie S. (2022). A characterization and an evolutionary and a pathogenicity analysis of reassortment H3N2 avian influenza virus in South China in 2019–2020. *Viruses*.

[22] Soda K., Kashiwabara M., Miura K. (2020). Characterization of H3 subtype avian influenza viruses isolated from poultry in Vietnam. *Virus Genes*.

[23] Zhang Y., Zhao C., Hou Y. (2021). Pandemic threat posed by H3N2 avian influenza virus. *Science China Life Sciences*.

[24] Zhang Q., Shi J., Deng G. (2013). H7N9 influenza viruses are transmissible in ferrets by respiratory droplet. *Science*.

[25] Yang Z.-F., Mok C. K. P., Peiris J. S. M., Zhong N.-S. (2015). Human Infection with a novel avian influenza A (H5N6) virus. *The New England Journal of Medicine*.

[26] Katoh K., Standley D. M. (2013). MAFFT multiple sequence alignment software version 7: improvements in performance and usability. *Molecular Biology and Evolution*.

[27] Gu W., Shi J., Cui P. (2022). Novel H5N6 reassortants bearing the clade 2.3.4.4b HA gene of H5N8 virus have been detected in poultry and caused multiple human infections in China. *Emerging Microbes & Infections*.

[28] Meng F., Chen Y., Song Z. (2023). Continued evolution of the Eurasian avian-like H1N1 swine influenza viruses in China. *Science China Life Sciences*.

[29] Reed L. J., Muench H. (1938). A simple method of estimating fifty per cent endpoints. *American Journal of Epidemiology*.

[30] Shi J., Deng G., Kong H. (2017). H7N9 virulent mutants detected in chickens in China pose an increased threat to humans. *Cell Research*.

[31] Xu R., McBride R., Nycholat C. M., Paulson J. C., Wilson I. A. (2012). Structural characterization of the hemagglutinin receptor specificity from the 2009 H1N1 influenza pandemic. *Journal of Virology*.

[32] Wang Z., Yang H., Chen Y. (2017). A single-amino-acid substitution at position 225 in hemagglutinin alters the transmissibility of eurasian avian-like H1N1 swine influenza virus in guinea pigs. *Journal of Virology*.

[33] Kong X., Guan L., Shi J. (2021). A single-amino-acid mutation at position 225 in hemagglutinin attenuates H5N6 influenza virus in mice. *Emerging Microbes & Infections*.

[34] Gambaryan A. S., Matrosovich T. Y., Philipp J. (2012). Receptor-binding profiles of H7 subtype influenza viruses in different host species. *Journal of Virology*.

[35] Xiong X., Martin S. R., Haire L. F. (2013). Receptor binding by an H7N9 influenza virus from humans. *Nature*.

[36] Mair C. M., Ludwig K., Herrmann A., Sieben C. (2014). Receptor binding and pH stability—how influenza A virus hemagglutinin affects host-specific virus infection. *Biochimica et Biophysica Acta (BBA) - Biomembranes*.

[37] Srinivasan K., Raman R., Jayaraman A., Viswanathan K., Sasisekharan R. (2013). Quantitative characterization of glycan-receptor binding of H9N2 influenza A virus hemagglutinin. *PLoS ONE*.

[38] Gao Y., Zhang Y., Shinya K. (2009). Identification of amino acids in HA and PB2 critical for the transmission of H5N1 avian influenza viruses in a mammalian host. *PLoS Pathogens*.

[39] Yamada S., Suzuki Y., Suzuki T. (2006). Haemagglutinin mutations responsible for the binding of H5N1 influenza A viruses to human-type receptors. *Nature*.

[40] Imai M., Watanabe T., Hatta M. (2012). Experimental adaptation of an influenza H5 HA confers respiratory droplet transmission to a reassortant H5 HA/H1N1 virus in ferrets. *Nature*.

[41] Maines T. R., Chen L.-M., Van Hoeven N. (2011). Effect of receptor binding domain mutations on receptor binding and transmissibility of avian influenza H5N1 viruses. *Virology*.

[42] Matrosovich M., Tuzikov A., Bovin N. (2000). Early alterations of the receptor-binding properties of H1, H2, and H3 avian influenza virus hemagglutinins after their introduction into mammals. *Journal of Virology*.

[43] Medeiros R., Naffakh N., Manuguerra J.-C., van der Werf S. (2004). Binding of the hemagglutinin from human or equine influenza H3 viruses to the receptor is altered by substitutions at residue 193. *Archives of Virology*.

[44] Narasaraju T., Sim M. K., Ng H. H. (2009). Adaptation of human influenza H3N2 virus in a mouse pneumonitis model: insights into viral virulence, tissue tropism and host pathogenesis. *Microbes and Infection*.

[45] Li X., Shi J., Guo J. (2014). Genetics, receptor binding property, and transmissibility in mammals of naturally isolated H9N2 avian influenza viruses. *PLoS Pathogens*.

[46] Song J., Xu J., Shi J., Li Y., Chen H. (2015). Synergistic effect of S224P and N383D substitutions in the PA of H5N1 avian influenza virus contributes to mammalian adaptation. *Scientific Reports*.

[47] Xu G., Zhang X., Gao W. (2016). Prevailing PA mutation K356R in avian influenza H9N2 virus increases mammalian replication and pathogenicity. *Journal of Virology*.

[48] Xiao C., Ma W., Sun N. (2016). PB2-588 V promotes the mammalian adaptation of H10N8, H7N9 and H9N2 avian influenza viruses. *Scientific Reports*.

[49] Song J., Feng H., Xu J. (2011). The PA protein directly contributes to the virulence of H5N1 avian influenza viruses in domestic ducks. *Journal of Virology*.

[50] Hulse-Post D. J., Franks J., Boyd K. (2007). Molecular changes in the polymerase genes (PA and PB1) associated with high pathogenicity of H5N1 influenza virus in mallard ducks. *Journal of Virology*.

[51] Hatta M., Gao P., Halfmann P., Kawaoka Y. (2001). Molecular basis for high virulence of Hong Kong H5N1 influenza A viruses. *Science*.

[52] Zhu W., Feng Z., Chen Y. (2019). Mammalian-adaptive mutation NP-Q357K in Eurasian H1N1 swine influenza viruses determines the virulence phenotype in mice. *Emerging Microbes & Infections*.

[53] Zhang Y., Zhang Q., Gao Y. (2012). Key molecular factors in hemagglutinin and PB2 contribute to efficient transmission of the 2009 H1N1 pandemic influenza virus. *Journal of Virology*.

[54] Feng X., Wang Z., Shi J. (2016). Glycine at Position 622 in PB1 contributes to the virulence of H5N1 avian influenza virus in mice. *Journal of Virology*.

[55] Liu Q., Qiao C., Marjuki H. (2012). Combination of PB2 271A and SR polymorphism at positions 590/591 is critical for viral replication and virulence of swine influenza virus in cultured cells and *in vivo*. *Journal of Virology*.

[56] Ma S., Zhang B., Shi J. (2020). Amino acid mutations A286V and T437M in the nucleoprotein attenuate H7N9 viruses in mice. *Journal of Virology*.

[57] Kong H., Ma S., Wang J. (2019). Identification of key amino acids in the PB2 and M1 proteins of H7N9 influenza virus that affect its transmission in guinea pigs. *Journal of Virology*.

[58] Cheng J., Zhang C., Tao J., Li B., Shi Y., Liu H. (2018). Effects of the S42 residue of the H1N1 swine influenza virus NS1 protein on interferon responses and virus replication. *Virology Journal*.

[59] Jiao P., Tian G., Li Y. (2008). A single-amino-acid substitution in the NS1 protein changes the pathogenicity of H5N1 avian influenza viruses in mice. *Journal of Virology*.

[60] Dong G., Peng C., Luo J. (2015). Adamantane-resistant influenza A viruses in the world (1902–2013): frequency and distribution of M2 gene mutations. *PLoS ONE*.

[61] Durrant M. G., Eggett D. L., Busath D. D. (2015). Investigation of a recent rise of dual amantadine-resistance mutations in the Influenza A M2 sequence. *BMC Genetics*.

[62] Gao R., Cao B., Hu Y. (2013). Human infection with a novel avian-origin influenza A (H7N9) virus. *The New England Journal of Medicine*.

[63] Cui H., Shi Y., Ruan T. (2016). Phylogenetic analysis and pathogenicity of H3 subtype avian influenza viruses isolated from live poultry markets in China. *Scientific Reports*.

[64] Lindh E., Ek-Kommonen C., Isomursu M. (2017). Genetic characterization of H13 and H16 Influenza A viruses in gulls (*Larus* Spp.) with clinically severe disease and concurrent circovirus infection. *Journal of Wildlife Diseases*.

[65] Li Y., Li M., Tian J. (2020). Characteristics of the first H16N3 subtype influenza A viruses isolated in western China. *Transboundary and Emerging Diseases*.

[66] Wang Z.-J., Kikutani Y., Nguyen L. T. (2018). H13 influenza viruses in wild birds have undergone genetic and antigenic diversification in nature. *Virus Genes*.

[67] Jing J., Wang L., Wang G. (2021). A human infection case with avian-origin H10N3 influenza virus. *Quantitative Imaging in Medicine and Surgery*.

[68] Chen H. Y., Yuan H., Gao R. (2014). Clinical and epidemiological characteristics of a fatal case of avian influenza A H10N8 virus infection: a descriptive study. *The Lancet*.

[69] Shi J., Zeng X., Cui P., Yan C., Chen H. (2023). Alarming situation of emerging H5 and H7 avian influenza and effective control strategies. *Emerging Microbes & Infections*.

[70] Bao P., Liu Y., Zhang X. (2022). Human infection with a reassortment avian influenza A H3N8 virus: an epidemiological investigation study. *Nature Communications*.

[71] Stevens J., Blixt O., Chen L.-M., Donis R. O., Paulson J. C., Wilson I. A. (2008). Recent avian H5N1 viruses exhibit increased propensity for acquiring human receptor specificity. *Journal of Molecular Biology*.

[72] Xu Q., Wang W., Cheng X., Zengel J., Jin H. (2010). Influenza H1N1 A/Solomon Island/3/06 virus receptor binding specificity correlates with virus pathogenicity, antigenicity, and immunogenicity in ferrets. *Journal of Virology*.

[73] de Graaf M., Fouchier R. A. M. (2014). Role of receptor binding specificity in influenza A virus transmission and pathogenesis. *The EMBO Journal*.

[74] Ibricevic A., Pekosz A., Walter M. J. (2006). Influenza virus receptor specificity and cell tropism in mouse and human airway epithelial cells. *Journal of Virology*.

[75] Li Q., Wang X., Sun Z. (2015). Adaptive mutations in PB2 gene contribute to the high virulence of a natural reassortant H5N2 avian influenza virus in mice. *Virus Research*.

[76] Yu Z., Sun W., Zhang X. (2017). Multiple amino acid substitutions involved in the virulence enhancement of an H3N2 avian influenza A virus isolated from wild waterfowl in mice. *Veterinary Microbiology*.

[77] Liang L., Jiang L., Li J. (2019). Low polymerase activity attributed to PA drives the acquisition of the PB2 E627K mutation of H7N9 avian influenza virus in mammals. *mBio*.

