# Supplementary material for: Genetic and Biological Characterization of H3N2 Avian Influenza Viruses Isolated from Poultry Farms in China between 2019 and 2021
Source: Transbound Emerg Dis. 2023 Jul 26;2023:8834913. doi: 10.1155/2023/8834913 (PMC12016730; doi:10.1155/2023/8834913)
Supplement: Supplementary 1 — Accession number of the reference sequences in the phylogenetic tree. [file 8834913.f1.pdf]

Table S1. Accession number of the reference sequences in the phylogenetic tree

Virus_name	Segment	Database	Accession number	Accessed Date
A/duck/Guangdong/F1172/2018(H3N2)	HA	GISAID	EPI_ISL_364305	20220605
A/duck/China/Influenza_A_virus/2019	HA	GISAID	EPI_ISL_6781438	20220605
A/wild_birds/Hubei/137/2014(H3N8)	HA	GISAID	EPI_ISL_505079	20220605
A/muscovy_duck/Vietnam/LBM348/2013(H3N8	HA	GISAID	EPI_ISL_181118	20220605
A/canine/Zhejiang/S34/2015(H3N8)	HA	GISAID	EPI_ISL_309172	20220605
A/chicken/Ganzhou/GZ43/2016(H3N2)	HA	GISAID	EPI_ISL_252831	20220605
A/Common_Teal/Amur_region/31b/2019(H3N6)	HA	GISAID	EPI_ISL_400266	20220605
A/long/tailed_duck/Wisconsin/16OS4632/2016(H3N8)	HA	GISAID	EPI_ISL_294164	20220605
A/northern_shoveler/Mongolia/899V/2009(H3N6)	HA	GISAID	EPI_ISL_161004	20220605
A/Duck/Bangladesh/BDADAI/3084/2019(H3N8)	HA	GISAID	EPI_ISL_6795370	20220605
A/Common_Teal/Omsk_Region/54/2019(H3N8)	HA	GISAID	EPI_ISL_400271	20220605
A/northern_pintail/Novosibirsk_region/3289k/2020(H3I	HA	GISAID	EPI_ISL_1184532	20220605
A/green/winged_teal/Alaska/AK20-528/2020(H3N8)	HA	GISAID	EPI_ISL_14767406	20220605
A/northern_pintail/Alaska/362/2013(H3N8)	HA	GISAID	EPI_ISL_257280	20220605
A/American_green/winged_teal/Alaska/98313/2008(H3I	HA	GISAID	EPI_ISL_234707	20220605
A/American_Green/Winged_Teal/Alaska/19TL00145/2(	HA	GISAID	EPI_ISL_4056504	20220605
A/duck/Fujian/SD063/2017(H3N3)	HA	GISAID	EPI_ISL_285381	20220605
A/duck/Hunan/161/2015(H3N6)	HA	GISAID	EPI_ISL_221746	20220605
A/duck/Hubei/ZYSYF12/2015(H3N6)	HA	GISAID	EPI_ISL_252812	20220605
A/duck/Guangdong/F138/2017(H3N2)	HA	GISAID	EPI_ISL_332678	20220605
A/chicken/Guangdong/F117/2018(H3N8)	HA	GISAID	EPI_ISL_364306	20220605
A/duck/China/322D22/2018(H3N2)	HA	GISAID	EPI_ISL_6780603	20220605
A/duck/Guangxi/175D12/2014(H3N6)	HA	GISAID	EPI_ISL_190031	20220605
A/chicken/Guangdong/F1201/2021(H3N8)	HA	GISAID	EPI_ISL_12949914	20220605
A/chicken/Fujian/F0112/2022(H3N8)	HA	GISAID	EPI_ISL_12946278	20220605
A/Henan/4/10/2022(H3N8)	HA	GISAID	EPI_ISL_12277126	20220605
A/Changsha/1000/2022(H3N8)	HA	GISAID	EPI_ISL_12703722	20220605
A/chicken/Vietnam/G14/2008(H3N8)	HA	GISAID	EPI_ISL_89570	20220605
A/white/backed_munia/Hong_Kong/4519/200	HA	GISAID	EPI_ISL_73977	20220605
A/mallard/Jiangxi/2-5/2010(H3N2)	HA	GISAID	EPI_ISL_167462	20220605
A/Aichi/2/1968(H3N2)	NA	Genbank	CY121119	20220610
A/chicken/China/2040/2020(H9N2)	NA	Genbank	ON329030	20220610
A/chicken/Ganzhou/GZ43/2016(H3N2)	NA	Genbank	KY415723	20220610
A/chicken/Guangxi/165C7/2014(H3N2)	NA	Genbank	KT022318	20220610
A/chicken/Hebei/045/2020(H9N2)	NA	Genbank	MZ895220	20220610
A/chicken/Hunan/02.06_YYGK11J3-OC/2018(H9N2)	NA	Genbank	MW099489	20220610
A/chicken/Shandong/017/2019(H9N2)	NA	Genbank	MZ702981	20220610
A/chicken/Shandong/105/2021(H9N2)	NA	Genbank	MZ703132	20220610
A/chicken/South Korea/SL20/2020(H9N2)	NA	Genbank	OP247634	20220610
A/chicken/Zhejiang/13163/2016(H9N2)	NA	Genbank	MW965672	20220610
A/duck/China/322D22/2018(H3N2)	NA	Genbank	MN443578	20220610
A/duck/China/F1473-2/2016(H6N2)	NA	Genbank	MT827989	20220610
A/duck/China/Influenza_A_virus/2019(H3N2)	NA	Genbank	OK104804	20220610
A/duck/Fujian/11.26_FZHX0195-O/2018(H9N2)	NA	Genbank	MW100413	20220610
A/duck/Guangxi/293D21/2017(H1N2)	NA	Genbank	MH667666	20220610
A/duck/Hubei/ZYSYG3/2015(H6N2)	NA	Genbank	KY415697	20220610
A/duck/Hunan/121/2014(H3N2)	NA	Genbank	KX121216	20220610
A/duck/Hunan/139/2014(H3N2)	NA	Genbank	KX121232	20220610
A/duck/Jiangxi/S21055/2012(H4N2)	NA	Genbank	KU161021	20220610
A/duck/Jiangxi/S3261/2009(H4N2)	NA	Genbank	KU161037	20220610
A/duck/Mongolia/296/2019(H4N2)	NA	Genbank	MT020168	20220610
A/duck/Tottori/311215/2020(H5N2)	NA	Genbank	LC656335	20220610
A/duck/Vietnam/HN5001/2018(H3N2)	NA	Genbank	MW936013	20220610
A/duck/Zhejiang/6D7/2013(H3N2)	NA	Genbank	KJ439872	20220610
A/duck/Zhejiang/727041/2014(H5N2)	NA	Genbank	KU042807	20220610
A/duck/Zhejiang/727042/2014(H6N2)	NA	Genbank	KT423163	20220610
A/Greylag Goose/South Korea/SW21/2021(H9N2)	NA	Genbank	OP268459	20220610
A/Hong_Kong/1/68(H3N2)	NA	Genbank	AF348184	20220610
A/mallard/Jiangxi/2-5/2010(H3N2)	NA	Genbank	CY098272	20220610
A/Muscovy duck/Vietnam/HN5609/2019(H9N2)	NA	Genbank	MW934936	20220610
A/New_York/73/2004(H3N2)	NA	Genbank	CY008518	20220610
A/Partridge/China/Q032/2013(H6N2)	NA	Genbank	MH592324	20220610
A/swine/Hong_Kong/NS2810/2011(H3N2)	NA	Genbank	KM028553	20220610
A/swine/Kansas/A02245216/2019(H3N2)	NA	Genbank	MN447257	20220610
A/swine/Korea/CAS05/2004(H3N2)	NA	Genbank	EU798829	20220610
A/white-backed_munia/Hong_Kong/4519/2009(H3N2)	NA	Genbank	AB557632	20220610
A/duck/Vietnam/HN6425/2020(H3N2)	PB2	Genbank	MW873031	20220610
A/duck/Zhejiang/S4489/2014(H7N9)	PB2	Genbank	MF630530	20220610

A/wild_duck/South_Korea/KNU18-91/2018(H5N3)	PB2	Genbank	MN493049	20220610
A/duck/Vietnam/HN5894/2019(H4N6)	PB2	Genbank	MW935149	20220610
A/Wild_Duck/South_Korea/KNU2020-101/2020(H9N2	PB2	Genbank	OK255519	20220610
A/duck/Jiangsu/SE0261/2018(H5N3)	PB2	Genbank	MN171447	20220610
A/duck/Zhejiang/6D7/2013(H3N2)	PB2	Genbank	KJ439817	20220610
A/Yangzhou/125/2022(H5N6)	PB2	Genbank	OP209766	20220610
A/duck/Wenzhou/YJYF78/2015(H1N4)	PB2	Genbank	KU143554	20220610
A/duck/China/322D22/2018(H3N2)	PB2	Genbank	MN443573	20220610
A/chicken/Guangxi/165C7/2014(H3N2)	PB2	Genbank	KT022322	20220610
A/duck/Vietnam/HN6479/2020(H3N2)	PB2	Genbank	MW872903	20220610
A/duck/Bangladesh/41847/2019(H10N2)	PB2	Genbank	MW466259	20220610
A/duck/Mongolia/961/2019(H3N8)	PB2	Genbank	MT020275	20220610
A/duck/Jiangxi/29344/2013(H7N3)	PB2	Genbank	KP416908	20220610
A/duck/Hubei/ZYSYF2/2015(H3N6)	PB2	Genbank	KY415878	20220610
A/Wild_Duck/South_Korea/KNU2020-74/2020(H3N8)	PB2	Genbank	OK235637	20220610
A/duck/Hainan/11.29_HKPL006-C/2017(H6N6)	PB2	Genbank	MW107813	20220610
A/Muscovy_duck/Vietnam/HN5693/2019(H4N2)	PB2	Genbank	MW935113	20220610
A/mallard/Jiangxi/37/2014(H7N3)	PB2	Genbank	MZ321811	20220610
A/Aichi/2/1968(H3N2)	PB2	Genbank	CY121124	20220610
A/Anas platyrhynchos/South Korea/JB29-91-95/2019(H	PB2	Genbank	MW126486	20220610
A/chicken/Ganzhou/GZ43/2016(H3N2)	PB2	Genbank	KY415899	20220610
A/chicken/Zhejiang/51043/2015(H1N9)	PB2	Genbank	KY971057	20220610
A/duck/Guangxi/293D21/2017(H1N2)	PB2	Genbank	MH667661	20220610
A/Hong_Kong/1/68(H3N2)	PB2	Genbank	AF348170	20220610
A/Muscovy_duck/Vietnam/HN5629/2019(H3N2)	PB2	Genbank	MW935304	20220610
A/New_York/73/2004(H3N2)	PB2	Genbank	CY008523	20220610
A/swine/Hong_Kong/NS2810/2011(H3N2)	PB2	Genbank	KM028548	20220610
A/swine/Korea/CAS05/2004(H3N2)	PB2	Genbank	EU798929	20220610
A/duck/Mongolia/709/2015(H10N7)	PB1	Genbank	LC121434	20220612
A/duck/China/322D22/2018(H3N2)	PB1	Genbank	MN443574	20220612
A/duck/Guangxi/175D12/2014(H3N6)	PB1	Genbank	KR919744	20220612
A/canine/Zhejiang/S34/2015(H3N8)	PB1	Genbank	MH018580	20220612
A/Muscovy_duck/Vietnam/HN5901/2019(H4N6)	PB1	Genbank	MW934691	20220612
A/duck/Vietnam/HN5474/2019(H3N2)	PB1	Genbank	MW935320	20220612
A/duck/Southern_China/04/2017(H7N9)	PB1	Genbank	MH114020	20220612
A/duck/Japan/AQ-HE103/2015(H1N2)	PB1	Genbank	LC278299	20220612
A/duck/Fujian/SD208/2017(H7N9)	PB1	Genbank	MH209513	20220612
A/duck/Jiangxi/11.17_NCNP48Y2-C/2016(H5N6)	PB1	Genbank	MW108622	20220612
A/duck/Hainan/Q221/2012(H1N2)	PB1	Genbank	KP658089	20220612
A/duck/Zhejiang/6DK19-MA/2013(H5N2)	PB1	Genbank	KX714304	20220612
A/duck/Jiangxi/S21046/2012(H4N2)	PB1	Genbank	KU161009	20220612
A/duck/Jiangxi/5465/2014(H7N3)	PB1	Genbank	KP417023	20220612
A/chicken/Zhejiang/528127/2016(H5N6)	PB1	Genbank	MW485697	20220612
A/duck/Zhejiang/6D20/2013(H10N2)	PB1	Genbank	KP063195	20220612
A/wild_duck/South_Korea/KNU18-91/2018(H5N3)	PB1	Genbank	MN493050	20220612
A/duck/Mongolia/MN2018-224/2018(H4N8)	PB1	Genbank	MW419965	20220612
A/Taiga_bean_goose/South_Korea/JB36-65/2019(H10N	PB1	Genbank	MW493150	20220612
A/duck/India/11CL01/2014(H6N2)	PB1	Genbank	KU598233	20220612
A/duck/Hokkaido/56/2017(H12N2)	PB1	Genbank	MK592491	20220612
A/chicken/Sichuan/k141/2017(H5N6)	PB1	Genbank	MH715337	20220612
A/duck/Mongolia/154/2015(H1N2)	PB1	Genbank	LC121274	20220612
A/duck/Hunan/139/2014(H3N2)	PB1	Genbank	KX121228	20220612
A/Aichi/2/1968(H3N2)	PB1	Genbank	CY121123	20220612
A/chicken/Ganzhou/GZ43/2016(H3N2)	PB1	Genbank	KY415855	20220612
A/chicken/Guangxi/165C7/2014(H3N2)	PB1	Genbank	KT022321	20220612
A/chicken/Zhejiang/102619/2016(H10N8)	PB1	Genbank	MG366496	20220612
A/duck/Bangladesh/38297/2019(H11N3)	PB1	Genbank	MT090432	20220612
A/duck/Guangxi/293D21/2017(H1N2)	PB1	Genbank	MH667662	20220612
A/duck/Hunan/161/2015(H3N6)	PB1	Genbank	KX121260	20220612
A/duck/Hunan/7/2015(H3N6)	PB1	Genbank	KX121268	20220612
A/duck/Jiangxi/15846/2013(H10N3)	PB1	Genbank	KP285475	20220612
A/duck/Minnesota/1375/1981(H1N1)	PB1	Genbank	CY014737	20220612
A/duck/Shantou/168/2007(H6N8)	PB1	Genbank	CY109720	20220612
A/duck/Wisconsin/3078/1986(H6N2)	PB1	Genbank	CY179481	20220612
A/duck/Zhejiang/6D7/2013(H3N2)	PB1	Genbank	KJ439828	20220612
A/Hong_Kong/1/68(H3N2)	PB1	Genbank	AF348172	20220612
A/duck/Fujian/13/2013(H1N8)	PA	Genbank	KP658053	20220612
A/duck/Hokkaido/W165/2015(H11N6)	PA	Genbank	LC339618	20220612
A/chicken/Fujian/C1161/2013(H9N2)	PA	Genbank	KM113268	20220612
A/wild_bird_/Shandong/4870/2019(H9N2)	PA	Genbank	MZ502901	20220612

A/Anseriformes/Anhui/L1/2014(H3N3)	PA	Genbank	MN148455	20220612
A/environment/Fujian/FJ1273/2014(H1N2)	PA	Genbank	KP658046	20220612
A/duck/Vietnam/HU9-194/2018(H6N6)	PA	Genbank	LC497096	20220612
A/duck/Zhejiang/6D20/2013(H10N2)	PA	Genbank	KP063196	20220612
A/duck/Zhejiang/6D7/2013(H3N2)	PA	Genbank	KJ439839	20220612
A/duck/Shantou/7904/2006(H6N2)	PA	Genbank	CY109425	20220612
A/duck/Shanghai/SD016/2015(H7N9)	PA	Genbank	MF630508	20220612
A/Duck/Mongolia/2019-496/2019(H1N1)	PA	Genbank	OK559470	20220612
A/mallard/Shanghai/JDS120662/2018(H10N4)	PA	Genbank	MN049525	20220612
A/duck/Bangladesh/31227/2016(H6N2)	PA	Genbank	MG042241	20220612
A/ruddy_turnstone/King_Island/14395/2019(H12N5)	PA	Genbank	OL370918	20220612
A/duck/Vietnam/HN6269/2020(H4N8)	PA	Genbank	MW873185	20220612
A/duck/Cambodia/b0120501/2017(H7N3)	PA	Genbank	MN703076	20220612
A/duck/Japan/AQ-HE103/2015(H1N2)	PA	Genbank	LC278300	20220612
A/White-fronted_Goose/South_Korea/KNU2018-26/202	PA	Genbank	OK217235	20220612
A/duck/Japan/AQ-HE29-52/2017(H7N9)	PA	Genbank	LC374948	20220612
A/chicken/Zhejiang/7418/2015(H1N3)	PA	Genbank	KY971111	20220612
A/chicken/Zhejiang/8615/2016(H10N3)	PA	Genbank	MG366499	20220612
A/duck/Fujian/1761/2010(H10N3)	PA	Genbank	KU921398	20220612
A/duck/Guangxi/135D20/2013(H3N2)	PA	Genbank	KT022304	20220612
A/duck/Wenzhou/775/2013(H7N2)	PA	Genbank	KF260449	20220612
A/black-tailed_gull/Shandong/W1496/2020(H10N8)	PA	Genbank	OM373213	20220612
A/duck/Mongolia/703/2018(H12N5)	PA	Genbank	MK978991	20220612
A/mallard/South_Korea/34X-2/2021(H7N7)	PA	Genbank	MZ803127	20220612
A/Anas_platyrhynchos/South_Korea/JB31-69/2019(H11	PA	Genbank	MW093747	20220612
A/duck/Bangladesh/38827/2019(H11N3)	PA	Genbank	MT090537	20220612
A/Aichi/2/1968(H3N2)	PA	Genbank	CY121122	20220612
A/chicken/Ganzhou/GZ43/2016(H3N2)	PA	Genbank	KY415811	20220612
A/chicken/Guiyang/4059/2005(H5N1)	PA	Genbank	EF124691	20220612
A/Duck(Anas sp.)/Mongolia/MN18-141/2018(H2N8)	PA	Genbank	MW412627	20220612
A/duck/China/322D22/2018(H3N2)	PA	Genbank	MN443575	20220612
A/duck/Guangxi/GXd-2/2012(H1N2)	PA	Genbank	KF013939	20220612
A/duck/Hunan/161/2015(H3N6)	PA	Genbank	KX121261	20220612
A/duck/Huzhou/4268/2013(H7N7)	PA	Genbank	KP413931	20220612
A/duck/Mongolia/210/2018(H3N2)	PA	Genbank	MW188572	20220612
A/duck/Vietnam/HN5684/2019(H3N2)	PA	Genbank	MW934698	20220612
A/duck/Vietnam/HN6105/2020(H4N2)	PA	Genbank	MW873318	20220612
A/Hong_Kong/1/68(H3N2)	PA	Genbank	AF348174	20220612
A/Mallard(Anas platyrhynchos)/South Korea/KNU2019	PA	Genbank	MW386776	20220612
A/mallard/Ohio/42/1989(H10N6)	PA	Genbank	CY089562	20220612
A/New_York/73/2004(H3N2)	PA	Genbank	CY008521	20220612
A/duck/Hunan/199/2014(H3N8)	NP	Genbank	KX121247	20220612
A/duck/Guangxi/293D21/2017(H1N2)	NP	Genbank	MH667665	20220612
A/duck/Guangdong/E1/2012(H10N8)	NP	Genbank	JQ924789	20220612
A/duck/Fujian/7818/2007(H6N6)	NP	Genbank	CY109907	20220612
A/duck/China/322D22/2018(H3N2)	NP	Genbank	MN443577	20220612
A/Duck/Dongting/D76-1/2016(H5N7)	NP	Genbank	MF362104	20220612
A/chicken/Zhejiang/516100/2017(H10N3)	NP	Genbank	MG366514	20220612
A/wild_bird_/Shandong/11706/2019(H9N2)	NP	Genbank	MZ502871	20220612
A/mallard/South_Korea/JB19-19/2019(H6N8)	NP	Genbank	MW492918	20220612
A/duck/Zhejiang/D9/2013(H4N6)	NP	Genbank	KT589249	20220612
A/duck/Zhejiang/2/2011(H7N3)	NP	Genbank	JQ906577	20220612
A/duck/Vietnam/HN5001/2018(H3N2)	NP	Genbank	MW936015	20220612
A/greylag_goose/Changsha/CS-510/2013(H4N8)	NP	Genbank	KJ907576	20220612
A/chicken/Zhejiang/7490/2015(H1N3)	NP	Genbank	KY971168	20220612
A/Mallard(Anas_platyrhynchos)/South_Korea/KNU201	NP	Genbank	MW404535	20220612
A/Aichi/2/1968(H3N2)	NP	Genbank	CY121120	20220612
A/chicken/Guangxi/165C7/2014(H3N2)	NP	Genbank	KT022319	20220612
A/chicken/Zhejiang/102619/2016(H10N8)	NP	Genbank	MG366517	20220612
A/duck/Guangdong/S4040/2011(H4N2)	NP	Genbank	KU160892	20220612
A/duck/Hunan/7/2015(H3N6)	NP	Genbank	KX121271	20220612
A/duck/Jiangxi/22676/2013(H7N3)	NP	Genbank	KP416824	20220612
A/duck/Vietnam/HN5684/2019(H3N2)	NP	Genbank	MW934702	20220612
A/duck/Vietnam/HN6038/2019(H11N2)	NP	Genbank	MW873362	20220612
A/duck/Wisconsin/3086/1986(H6N2)	NP	Genbank	CY179494	20220612
A/environment/Fujian/EV01/2020(H11N3)	NP	Genbank	ON968460	20220612
A/Hong_Kong/1/68(H3N2)	NP	Genbank	AF348180	20220612
A/mallard/Ohio/181/1986(H3N1)	NP	Genbank	CY021432	20220612
A/New_York/73/2004(H3N2)	NP	Genbank	CY008519	20220612
A/wild_goose/dongting_lake/121/2018(H6N2)	NP	Genbank	MH727481	20220612

A/duck/Zhejiang/925091/2014(H4N2)	M	Genbank	KT589283	20220614
A/mallard/Alaska/327/2013(H3N8)	M	Genbank	KY130996	20220614
A/duck/Vietnam/HN5337/2019(H4N6)	M	Genbank	MW934747	20220614
A/duck/Zhejiang/77139/2014(H4N2)	M	Genbank	KT589278	20220614
A/duck/Guangdong/8.30_DGCP036-C/2017(H6N2)	M	Genbank	MW100982	20220614
A/duck/Guangdong/DGQTSJ147P/2015(H4N8)	M	Genbank	KY785664	20220614
A/duck/Guangxi/293D21/2017(H1N2)	M	Genbank	MH667667	20220614
A/duck/Zhejiang/6D20/2013(H10N2)	M	Genbank	KP063200	20220614
A/duck/Yuhuan/YH45/2016(H1N2)	M	Genbank	KY415671	20220614
A/duck/Shanghai/SH1/2013(H3N2)	M	Genbank	KM222559	20220614
A/duck/Guangxi/175D12/2014(H3N6)	M	Genbank	KR919747	20220614
A/duck/Hubei/ZYSYF4/2015(H3N6)	M	Genbank	KY415647	20220614
A/duck/Guangxi/SDY129/2014(H7N9)	M	Genbank	MF630448	20220614
A/duck/China/322D22/2018(H3N2)	M	Genbank	MN443579	20220614
A/chicken/Huzhou/3916/2013(H7N3)	M	Genbank	KP413761	20220614
A/muscovy_duck/Vietnam/LBM115/2012(H3N2)	M	Genbank	LC028107	20220614
A/Aichi/2/1968(H3N2)	M	Genbank	CY121118	20220614
A/Bean Goose(Anser fabalis)/South Korea/KNU2021-39	M	Genbank	ON505887	20220614
A/blue-winged teal/Guatemala/CIP049H105-15/2011(H	M	Genbank	KJ195679	20220614
A/chicken/Ganzhou/GZ43/2016(H3N2)	M	Genbank	KY415679	20220614
A/chicken/Guangxi/165C7/2014(H3N2)	M	Genbank	KT022324	20220614
A/duck/Hunan/161/2015(H3N6)	M	Genbank	KX121265	20220614
A/duck/Mongolia/398/2018(H3N8)	M	Genbank	MW188616	20220614
A/duck/Mongolia/496/2010(H3N3)	M	Genbank	LC367397	20220614
A/duck/Mongolia/619/2019(H3N6)	M	Genbank	MT020209	20220614
A/duck/Zhejiang/6D7/2013(H3N2)	M	Genbank	KJ439883	20220614
A/duck/Zhejiang/727D2/2013(H11N3)	M	Genbank	KX028835	20220614
A/Hong_Kong/1/68(H3N2)	M	Genbank	AF348189	20220614
A/mallard/Alberta/177/2004(H7N9)	M	Genbank	KX827374	20220614
A/New_York/73/2004(H3N2)	M	Genbank	CY008517	20220614
A/Pigeon/Longquan/LQ67/2016(H2N8)	M	Genbank	KY415672	20220614
A/spot-billed duck/Ningxia/YG83/2017(H5N6)	M	Genbank	MH283040	20220614
A/turkey/Illinois/2004(H3N2)	M	Genbank	EF551048	20220614
A/turkey/Ontario/31232/2005(H3N2)	M	Genbank	DQ470001	20220614
A/duck/Zhejiang/6D7/2013(H3N2)	NS	Genbank	KJ439894	20220614
A/duck/Shanghai/C84/2009(H3N2)	NS	Genbank	JX286595	20220614
A/duck/Mongolia/MN18-14/2018(H3N8)	NS	Genbank	MW487356	20220614
A/duck/Hubei/ZYSYF21/2015(H3N6)	NS	Genbank	KY415920	20220614
A/wild_waterbird/Western_Australia/AS19-1358-170/20	NS	Genbank	OL371907	20220614
A/duck/Guangdong/8.30_DGCP036-C/2017(H6N2)	NS	Genbank	MW101937	20220614
A/duck/Bangladesh/39397/2019(H10N3)	NS	Genbank	MT090628	20220614
A/wild_goose/dongting_lake/121/2018(H6N2)	NS	Genbank	MH727484	20220614
A/Wild_duck/South_Korea/KNU2020-31/2020(H1N1)	NS	Genbank	OK217178	20220614
A/wild_duck/South_Korea/KNU18-107/2018(H7N7)	NS	Genbank	MN483257	20220614
A/pintail/Taiwan/WB2478/2017(H1N3)	NS	Genbank	MN988825	20220614
A/Muscovy_duck/Vietnam/HN4929/2018(H9N1)	NS	Genbank	MW935904	20220614
A/mallard/Jiangxi/G98/2014(H3N8)	NS	Genbank	MN473217	20220614
A/common_teal/Shanghai/JDS110203/2019(H12N8)	NS	Genbank	MN795771	20220614
A/wild_bird_/Shandong/11706/2019(H9N2)	NS	Genbank	MZ502874	20220614
A/mallard/South_Korea/JB22-3/2019(H5N3)	NS	Genbank	MW493135	20220614
A/duck/Mongolia/374/2018(H4N6)	NS	Genbank	MW188609	20220614
A/duck/Guangdong/S1123/2012(H4N2)	NS	Genbank	KU160879	20220614
A/chicken/Jiangsu/YC/2015(H4N8)	NS	Genbank	KY785671	20220614
A/duck/Shantou/2962/2007(H6N6)	NS	Genbank	CY109774	20220614
A/duck/Jiangxi/15867/2013(H10N3)	NS	Genbank	KP285497	20220614
A/duck/China/F1473-2/2016(H6N2)	NS	Genbank	MT828327	20220614
A/duck/Jiangxi/20151/2013(H7N3)	NS	Genbank	KP416720	20220614
A/Aichi/2/1968(H3N2)	NS	Genbank	CY121121	20220614
A/chicken/Ganzhou/GZ43/2016(H3N2)	NS	Genbank	KY415943	20220614
A/chicken/Guangxi/129/2013(H6N6)	NS	Genbank	KT266994	20220614
A/chicken/Guangxi/165C7/2014(H3N2)	NS	Genbank	KT022323	20220614
A/Duck(Anas sp.)/Mongolia/MN18-141/2018(H2N8)	NS	Genbank	MW412632	20220614
A/duck/Bangladesh/38292/2019(H2N2)	NS	Genbank	MT090541	20220614
A/duck/Bangladesh/38305/2019(H11N3)	NS	Genbank	MT090556	20220614
A/duck/Cambodia/C50W8M1/2018(H7N4)	NS	Genbank	MN703009	20220614
A/duck/Guangxi/135D20/2013(H3N2)	NS	Genbank	KT022307	20220614
A/duck/Guangxi/293D21/2017(H1N2)	NS	Genbank	MH667668	20220614
A/duck/Guizhou/5.24_ZYLJJ016-O/2018(H6N6)	NS	Genbank	MW101819	20220614
A/duck/Mongolia/496/2010(H3N3)	NS	Genbank	LC367398	20220614
A/duck/Vietnam/HN5684/2019(H3N2)	NS	Genbank	MW934696	20220614

A/Hong_Kong/1/68(H3N2)	NS	Genbank	AF348198	20220614
A/New_York/73/2004(H3N2)	NS	Genbank	CY008520	20220614
A/waterfowl/Korea/S353/2016(H11N9)	NS	Genbank	KX703023	20220614
A/wild waterbird/Western Australia/AS19-3999-1/2019(	NS	Genbank	OL371923	20220614
