# Supplementary material for: Genetic and Biological Characterization of H3N2 Avian Influenza Viruses Isolated from Poultry Farms in China between 2019 and 2021
Source: Transbound Emerg Dis. 2023 Jul 26;2023:8834913. doi: 10.1155/2023/8834913 (PMC12016730; doi:10.1155/2023/8834913)
Supplement: Supplementary 2 — Phylogenetic trees of the genes of H3N2 AIVs. The phylogenetic tree, HA (a), NA (b), PB2 (c), PB1 (d), PA (e), NP (f), M (g), and NS (h), was generated by using the neighbor-joining method and the MEGA 7.0 software package, with 1,000 bootstrap replicates. Sequences with a nucleotide identity of more than 95% were categorized into the same group. The viruses isolated in this study are colored in the phylogenetic trees; viruses in black were downloaded from available databases. The scale bar indicates the number of nucleotide substitutions per site. [file 8834913.f2.pdf]

Fig. S1. Phylogenetic trees of the genes of H3N2 avian influenza viruses.

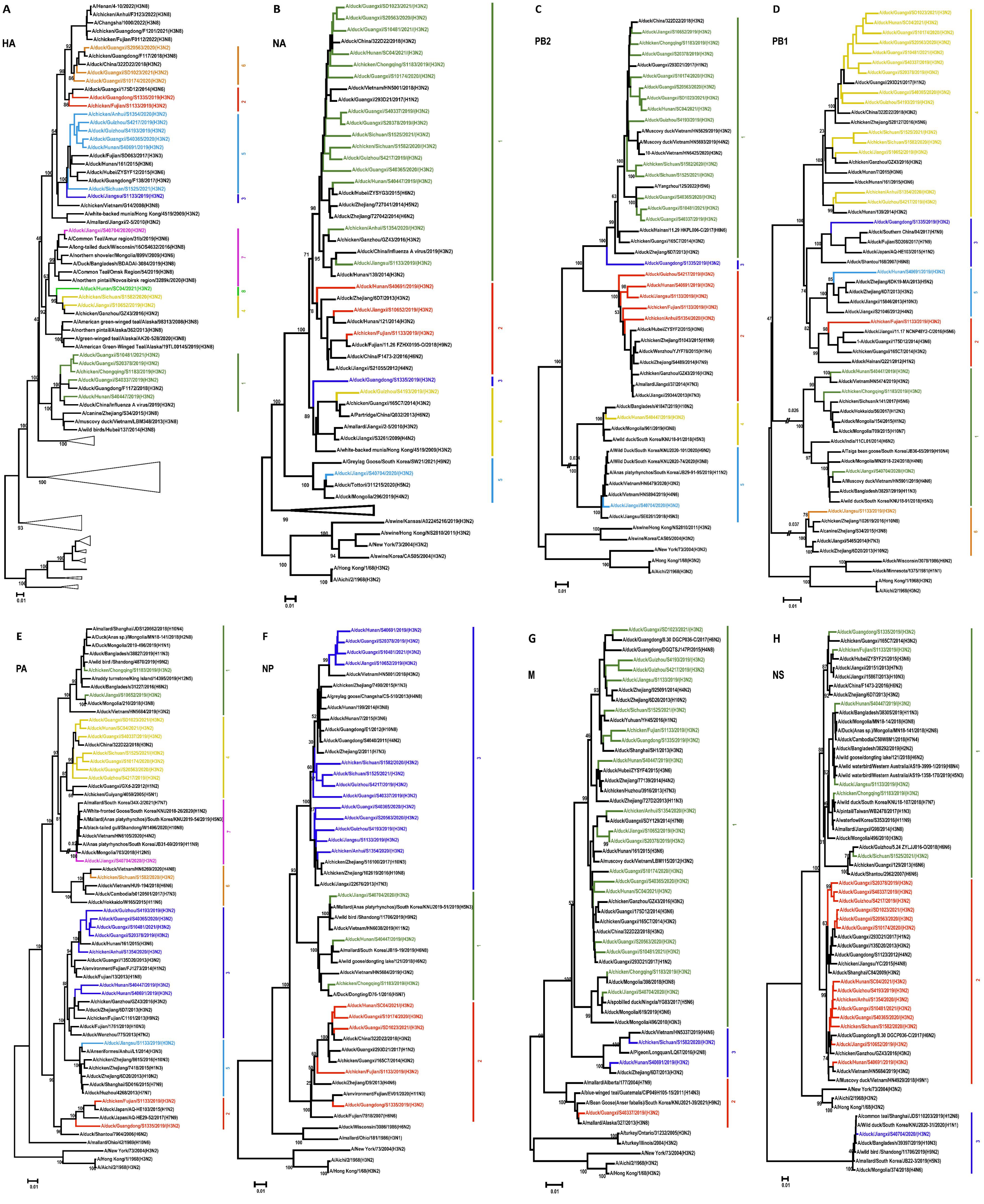

Fig. S1. Phylogenetic trees of the genes of H3N2 avian influenza viruses. The phylogenetic tree, HA (A), NA (B), PB2 (C), PB1 (D), PA (E), NP (F), M(G) and NS (H), was generated by using the neighbor-joining method and the MEGA 7.0 software package, with 1000 bootstrap replicates. Sequences with a nucleotide identity of more than 95% were categorized into the same group. The viruses isolated in this study are colored in the phylogenetic trees; viruses in black were downloaded from available databases. The scale bar indicates the number of nucleotide substitutions per site.
