# Supplementary material for: Genetic and Biological Characterization of H3N2 Avian Influenza Viruses Isolated from Poultry Farms in China between 2019 and 2021
Source: Transbound Emerg Dis. 2023 Jul 26;2023:8834913. doi: 10.1155/2023/8834913 (PMC12016730; doi:10.1155/2023/8834913)
Supplement: Supplementary 3 — Antigenic analysis of H3N2 AIVs isolated from 2019 to 2021 in China. [file 8834913.f3.pdf]

Table S2. Antigenic analysis of H3N2 avian influenza viruses isolated from 2019 to 2021 in China.

Virus	Hemagglutination Inhibition titers of chicken antiserum against different H3N2 viruses						
	A/duck/Jiangxi/S10652/2019	A/duck/Guangxi/S10481/2021	A/duck/Guizhou/S4193/2019	A/duck/Hunan/S40691/2019	A/chicken/Anhui/S1354/2020	A/duck/Guangxi/S40365/2020	A/duck/Hunan/SC04/2021
A/chicken/Chongqing/S1183/2019	128	256	128	128	64	128	256
A/chicken/Fujian/S1133/2019	64	256	512	128	512	256	512
A/duck/Guangdong/S1335/20	64	128	256	128	256	256	256
A/duck/Jiangsu/S1133/2019	128	256	256	256	128	256	512
A/duck/Jiangxi/S10652/2019	**256***	256	128	128	64	128	256
A/duck/Guangxi/S20378/2019	128	256	128	128	128	128	256
A/duck/Guangxi/S10481/2021	128	**512**	256	128	128	128	512
A/duck/Guangxi/S40337/2019	256	256	256	256	128	256	512
A/duck/Guizhou/S4193/2019	128	256	**512**	512	512	512	512
A/duck/Guizhou/S4217/2019	128	256	512	256	512	256	512
A/duck/Hunan/S40447/2019	256	512	512	256	128	256	512
A/duck/Hunan/S40691/2019	128	256	512	**256**	512	512	512
A/chicken/Anhui/S1354/2020	128	256	512	256	**512**	256	256
A/chicken/Sichuan/S1582/202	256	512	512	256	256	512	512
A/duck/Guangxi/S10174/2020	128	256	512	256	256	256	512
A/duck/Guangxi/SD1023/202	128	256	512	256	256	256	512
A/duck/Guangxi/S20563/2020	128	256	512	256	256	256	512
A/duck/Guangxi/S40365/2020	128	256	512	256	512	**512**	512
A/duck/Jiangxi/S40704/2020	64	256	256	256	256	256	128
A/duck/Hunan/SC04/2021	128	128	256	128	128	128	**512**
A/duck/Sichuan/S1525/2021	128	256	256	256	128	256	512

*Homologous titers are in Bold.
